# The Effect of Upadacitinib on Lipid Profile and Cardiovascular Events: A Meta-Analysis of Randomized Controlled Trials

**DOI:** 10.3390/jcm11236894

**Published:** 2022-11-22

**Authors:** Anastasios Makris, Fotios Barkas, Petros P. Sfikakis, Evangelos Liberopoulos, Aris P. Agouridis

**Affiliations:** 1 School of Medicine, European University Cyprus, Nicosia 2404, Cyprus; 2 Department of Hygiene & Epidemiology, Faculty of Medicine, School of Health Sciences, University of Ioannina, 45110 Ioannina, Greece; 3 First Department of Propaedeutic Medicine, Medical School, Laiko General Hospital, National and Kapodistrian University of Athens, 11527 Athens, Greece; 4 Department of Internal Medicine, German Oncology Center, Limassol 4108, Cyprus

**Keywords:** upadacitinib, ABT-494, Janus kinase inhibitors, cardiovascular disease, lipids, cholesterol

## Abstract

Background: Our aim was to systematically investigate the effect of upadacitinib, an oral JAK-1 selective inhibitor, on lipid profile and cardiovascular disease risk. Methods: PubMed, PubMed Central and ClinicalTrials.gov databases were searched for relevant randomized controlled trials (RCTs) up to 31 July 2022. We performed a qualitative synthesis of published RCTs to investigate the associations of upadacitinib with lipoprotein changes, along with a quantitative synthesis of MACE and mean lipoprotein changes where there were available data. Results: Nineteen RCTs were eligible for the present systematic review, which included 10,656 patients with a mean age of 51 years and a follow-up period of 12–52 weeks. Increases in low-density lipoprotein cholesterol (LDL-C) and high-density lipoprotein cholesterol (HDL-C) were noted upon upadacitinib administration (3–48 mg/day) in 15 studies, while the LDL-C:HDL-C ratio remained unchanged. The pooled analyses of three placebo-controlled RCTs (*n* = 2577) demonstrated that upadacitinib at 15 mg increased the LDL-C by 15.18 mg/dL (95% CI: 7.77–22.59) and HDL-C by 7.89 mg/dL (95% CI: 7.08–8.69). According to the pooled analysis of 15 placebo-controlled RCTs (*n* = 7695), upadacitinib had no effect on MACE (risk ratio, RR: 0.62; 95% CI: 0.24–1.60). A sub-analysis focusing on upadacitinib at 15 mg (12 studies, *n* = 5395) demonstrated similar results (RR: 0.67; 95% CI: 0.19–2.36). Conclusions: Treatment with upadacitinib increases both LDL-C and HDL-C levels. Nevertheless, upadacitinib had no significant effect on the cardiovascular disease risk during a ≤52-week follow-up.

## 1. Introduction

Following the discovery of the Janus kinase–signal transduction and activation of transcription (JAK-STAT) pathway and its roles in inflammation and neoplasia, a new class of drugs, the Janus kinase inhibitors (JAKinhibs), were introduced a decade ago. The advent of this new era was marked by the development of the pan-JAK inhibitor tofacitinib in 2011 [1]. These agents possess a variety of indications ranging from rheumatic to dermatologic and oncologic/hematologic conditions, as well as coronavirus disease (COVID)-19 [1]. The overall efficacy and safety of JAKinibs have been shown to be comparable to those of biological agents and even superior in certain circumstances, such as rheumatoid arthritis (RA) [1,2]. Their most common adverse events include infections (especially herpes zoster) and increases in cholesterol levels, as well as in muscle and liver enzymes. Although the evidence supports the notion that treatment with JAKinibs is associated with increases in cholesterol indices, their effect on cardiovascular disease risk appears neutral [3].

In the present systematic review, we focus on upadacitinib, or ABT-494 (AbbVie, North Chicago, IL, USA), an oral JAK-1 selective inhibitor licensed in 2019 and approved for RA, psoriatic arthritis (PsA), and ankylosing spondylitis (AS) and currently being tested for several other indications, such as inflammatory bowel disease, vasculitis, atopic dermatitis (AD), and hidradenitis suppurativa [2,4,5]. Herein, we systematically investigate the effects of upadacitinib therapy on lipoproteins and cardiovascular disease risk.

## 2. Materials and Methods

This systematic review was registered on PROSPERO (ID number: CRD42022289988) and adheres to the Preferred Reporting Items for Systematic Reviews and Meta-Analyses (PRISMA) 2020 statement (PRISMA Statement, Ottawa, ON, Canada) [6].

The primary outcome of the present study was the assessment of the impact of upadacitinib on lipid profile. The secondary outcome was the investigation of its effect on cardiovascular disease risk. The included studies assessed both the efficacy and safety of upadacitinib therapy and were selected on the basis of their available data on the relevant endpoints.

### 2.1. Search Strategy

A systematic search of the PubMed, PubMed Central and ClinicalTrials.gov databases was conducted in English up to July 2022. To identify relevant randomized controlled trials (RCTs), the search terms were: (“Upadacitinib” OR “Jak-1 inhibitor” OR “ABT-494”) AND (“Cholesterol” OR “Cardiovascular”). References from the retrieved articles were scrutinized to identify additional suitable studies.

### 2.2. Screening and Eligibility

The records were deduplicated by the use of the Zotero reference managing software. The eligibility screening process involved two steps: first, one author (A.M) excluded non-eligible studies after screening titles and abstracts and evaluated the remaining full texts for eligibility. In the second step, another author (A.P.A.) verified these decisions. Any disagreements were resolved upon discussion and joint examination of the proposed articles. The eligibility criteria were formed based on the PICOS (population, intervention, comparators/controls, outcomes, and study design) study question format, as follows (Supplementary Table S1):

Population: Patients receiving upadacitinib.

Intervention: Administration of upadacitinib, alone or in conjunction with other agents.

Comparators/controls: Placebo or any other intervention.

Outcomes: Effect on lipid profile (LDL-C, HDL-C, LDL-C:HDL-C) and/or major adverse cardiovascular events (i.e., myocardial infarction, stoke, and cardiovascular death).

Study design: Only RCTs were included (phase II–III).

Studies not meeting the eligibility criteria were excluded.

### 2.3. Data Extraction

Two authors (A.M. and A.P.A.) separately extracted data from the eligible studies. The data were subsequently added to an electronic document (an Excel spreadsheet) to avoid possible errors in data entry. Reviewer discrepancies were resolved, and a consensus was reached. The extracted data reported on the following variables: first author; country; DOI; study type; trial phase; total number of patients enrolled; population characteristics; intervention; comparator; duration of treatment; mean changes in LDL-C, HDL-C, and LDL-C:HDL-C ratio; adverse events (i.e., major adverse cardiovascular events (MACE)). For this meta-analysis, all relevant adverse events that were noted in the same patient were recorded as separate, while transient ischemic attacks were recorded as strokes. The abstraction of graphical data was performed by use of open-source software when needed.

### 2.4. Methodological Assessment of the Included Studies

For the assessment of the eligible randomized controlled trials, we used the revised Cochrane risk-of-bias tool for randomized trials (RoB-2). Based on this algorithm, studies are classified as ‘low-risk’, ‘high-risk’, or ‘with some concerns’ regarding bias. The following items were evaluated: generation of a random sequence; allocation sequence concealment; blinding; exclusion and participant loss; other possible sources of bias; and design-specific bias [7]. The risk of bias of each study was evaluated independently by A.M. and A.P.A., and discrepancies were resolved by reaching a consensus.

### 2.5. Data Analysis

Where sufficient information on the primary and secondary endpoints was obtainable based on a minimum of three trials, and the outcome measures were comparable, meta-analyses were performed, allowing for a quantitative analysis of the studies. Pooled estimations regarding outcomes were expressed as dichotomous for MACE, while mean LDL-C and HDL-C changes secondary to upadacitinib therapy were expressed as continuous. Meta-analyses were performed using a random effects model or a fixed effects model. For dichotomous data, pooled risk ratios (RR) and 95% confidence intervals (CΙs) were calculated, whereas mean differences were calculated for continuous data. Regarding MACE, a subgroup analysis for the dose of 15 mg was performed according to AE using 12 of the 15 studies included in the original MACE analysis. A statistical analysis was performed and forest plots were generated using the Review Manager (RevMan) Version 5.0 software (The Nordice Cochrane Center, The Cochrane Collaboration, Copenhagen, Denmark, 2008). A *p* < 0.05 was considered significant.

### 2.6. Heterogeneity Analysis

The existence of statistical heterogeneity among the included studies was assessed using the I^2^ test. The heterogeneity was considered low, moderate, or high if the I^2^ was 25%, 50%, or >75%, respectively. If the *p*-value was less than 0.10, the random effects model was adopted, and vice versa. The inter-trial heterogeneity was assessed using the Q test and the I^2^ statistic.

## 3. Results

### 3.1. Study Selection

**Figure 1:**
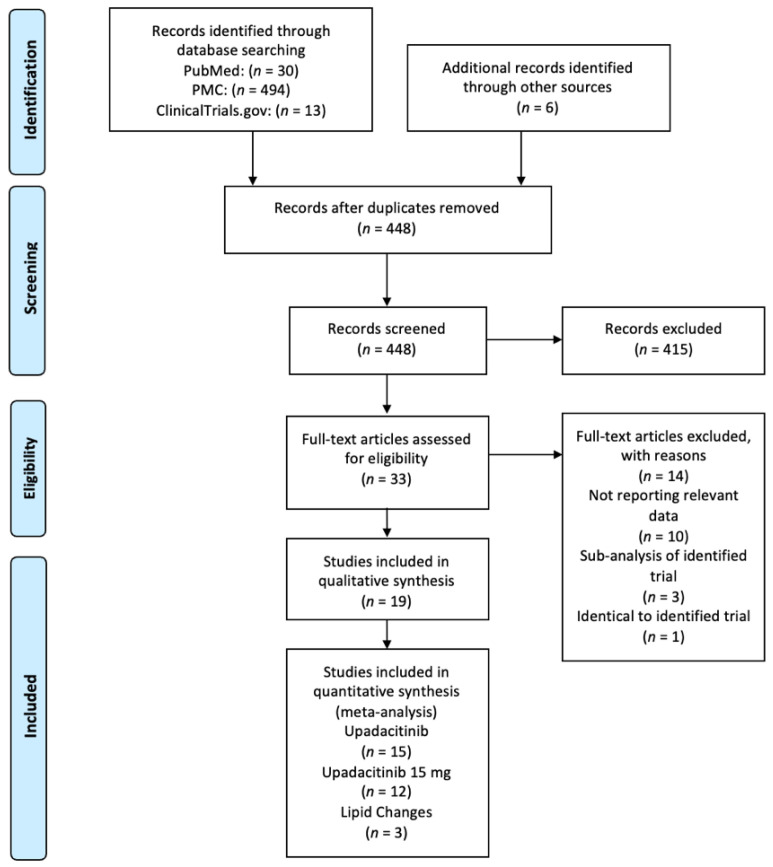
Literature and study selection performed by use of the PRISMA FLOWCHART.

In Figure 1, the PRISMA flowchart shows the study selection process. Using the appropriate search terms, we identified 543 records and, finally, 19 studies met the inclusion criteria [8,9,10,11,12,13,14,15,16,17,18,19,20,21,22,23,24,25,26]. The excluded studies [27,28,29,30,31,32,33,34,35,36,37,38,39,40], together with the reasons for their exclusion, are presented in Supplementary Table S2.

### 3.2. Study Characteristics

All identified trials were performed between 2016 and 2022 and were multicenter phase II or III randomized controlled trials. They included a total of 10,656 patients with a mean age of 51 years and a follow-up period ranging from 12 to 52 weeks. In total, 11 studies were conducted on patients with rheumatoid arthritis, along with 2 on patients with atopic dermatitis, 2 on patients with psoriatic arthritis, 3 on patients with spondyloarthritis (ankylosing spondylitis and non-radiographic axial spondyloarthritis), and 1 on patients with Crohn’s disease. Two studies, both conducted by Fleischmann et al., used the same set of patients [14,22]. Details of the included studies are presented in Table 1.

### 3.3. Study Outcomes

Results are summarized in Table 2. Fifteen studies showed increasing trends in both LDL-C and HDL-C, with LDL-C changes ranging from −1.28 to 27.84 mg/dL and HDL-C ranging from 1.93 to 18.56 mg/dL. As shown in Table 2, Sandborn et al. demonstrated a significant LDL-C increase in the 12 mg and 24 mg BID upadacitinib groups at 16 weeks compared with the placebo group, but not at 52 weeks (data on the 24 mg BID at 52 weeks were missing) [10]. In the same study, HDL-C increased significantly in the 12 mg upadacitinib BID group at both 16 and 52 weeks compared with placebo [10]. Furthermore, Yassky et al. showed significant increases in both LDL-C and HDL-C only in the upadacitinib 30 mg group when compared with the placebo group (Table 2) [21]. Lastly, Genovese et al. demonstrated a statistically significant LDL-C increase in all upadacitinib doses (i.e., 3, 6, 12, 18 mg BID and 24 mg QD) in comparison with placebo, while HDL-C changes were only significant in the upadacitinib 6 mg group [9].

**Figure 2:**
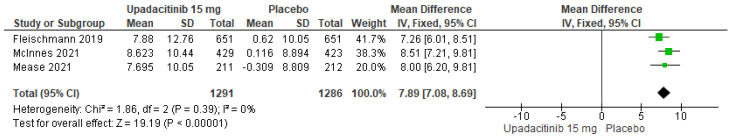
Forest plot of the comparison: upadacitinib vs. placebo. Outcome: high-density lipoprotein cholesterol (mg/dL) [17,20,22].

**Figure 3:**
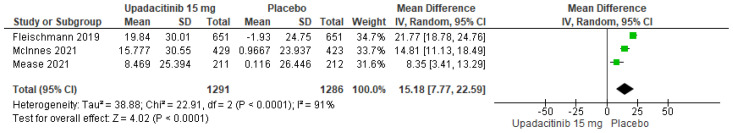
Forest plot of the comparison: upadacitinib vs. placebo. Outcome: low-density lipoprotein cholesterol (mg/dL) [17,20,22].

Three placebo-controlled RCTs (*n* = 2577) had available data to compare the effects of upadacitinib 15 mg on lipoproteins vs. placebo. The pooled analysis showed that treatment with upadacitinib at 15 mg increased HDL-C by 7.89 mg/dL (95% CI: 7.08–8.69; I^2^ = 0%, *p* = 0.39; Figure 2) and LDL-C by 15.18 mg/dL (95% CI: 7.77–22.59; I^2^ = 91%, *p* < 0.0001; Figure 3).

In total, 22 MACE were noted in patients who were treated with upadacitinib (*n* = 6219) vs. 11 in the control groups (*n* = 4437) during follow-up (≤52 weeks). As far as the dose of 15 mg [41] is concerned, 11 MACE were noted in the upadacitinib arm (*n* = 3533) compared to 4 MACE noted in the control group (*n* = 4294).

**Figure 4:**
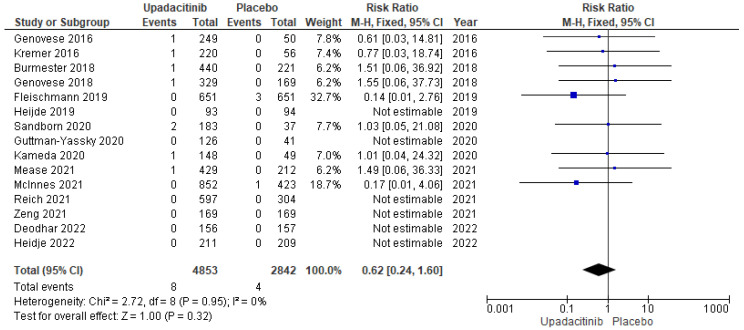
Forest plot of the comparison: upadacitinib vs. placebo. Outcome: major adverse cardiovascular events [8,9,10,11,12,15,16,17,20,21,22,23,24,25,26].

**Figure 5:**
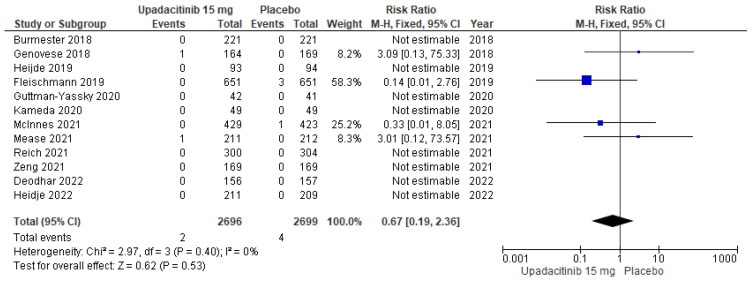
Forest plot of the comparison: upadacitinib 15 mg vs. placebo. Outcome: MACE (major adverse cardiovascular events) [11,12,13,14,15,16,17,20,21,22,23,24,25,26].

As shown in Figure 4, the pooled analysis of the 15 placebo-controlled RCTs (*n* = 7695) showed that upadacitinib had no effect on MACE risk (RR: 0.62; 95% CI: 0.24–1.60; I^2^ = 0, *p* = 0.95). Likewise, the pooled analysis of 12 studies (*n* = 5395) showed similar results for the dose of 15 mg (RR: 0.67; 95% CI: 0.19–2.36; I^2^ = 0, *p* = 0.40; Figure 5).

### 3.4. Risk of Bias Assessment

**Figure 6:**
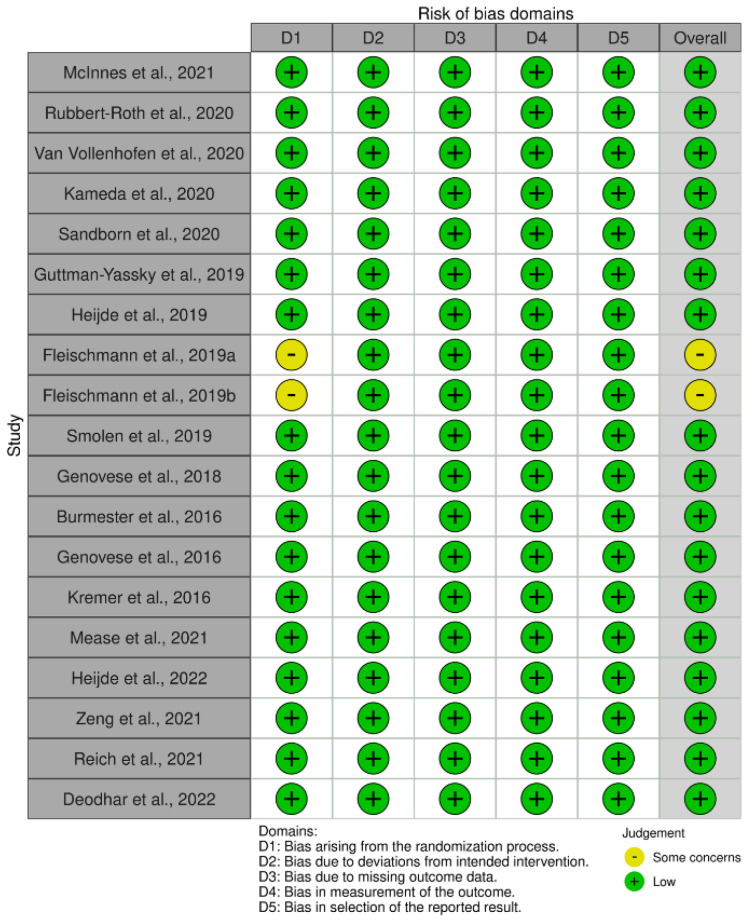
Traffic light plot for risk of bias assessment of the included studies using the revised Cochrane risk-of-bias tool for randomized trials (RoB-2) [8,9,10,11,12,13,14,15,16,17,18,19,20,21,22,23,24,25,26].

**Figure 7:**
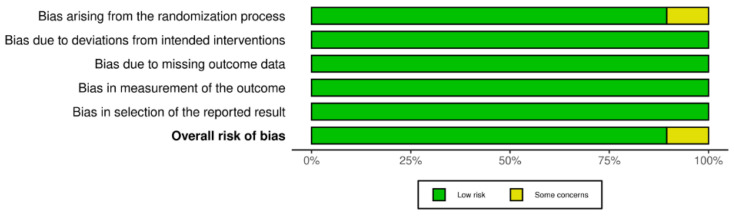
Summary plot for risk of bias assessment of the included studies using the revised Cochrane risk-of-bias tool for randomized trials (RoB-2) [8,9,10,11,12,13,14,15,16,17,18,19,20,21,22,23,24,25,26].

The bias risk was assessed using the revised Cochrane risk-of-bias tool for randomized trials 2 (RoB-2) (Figure 6 and Figure 7). Based on this tool, the eligible studies were screened for potential bias arising from five different domains, namely, randomization; protocol deviation; data availability; outcome measurement method; and selection of reported results. Out of the 19 studies assessed, only 2 studies, both conducted by Fleischmann et al. [14,22], were judged to have ‘some concerns’ regarding bias due to problems in the randomization process. All the other trials were judged to have a low bias risk.

## 4. Discussion

To the best of our knowledge, this is the first systematic review and meta-analysis assessing the effects of upadacitinib on lipid profile and MACE across different disease populations. LDL-C and HDL-C increases seemed to be consistently greater in the upadacitinib groups vs. comparators across the included studies, whereas the LDL-C:HDL-C ratio did not significantly change. This meta-analysis shows that treatment with the approved dose of 15 mg is associated with significant increases in both LDL-C and HDL-C compared with placebo. The pooled analysis of the 15 placebo-controlled RCTs demonstrated that upadacitinib had no effect on the risk of MACE.

Our results are in line with those of previous studies [42,43]. A recent systematic review and meta-analysis investigating the effects of JAKinhibs on the lipid profiles of 6697 patients with RA, which included only two studies on upadacitinib, showed that most JAKinibs increased both LDL-C and HDL-C levels by 8.11 mg/dL (95% CI: 6.65–9.58) and 11.37 mg/dL (95% CI: 7.84–14.91), respectively. No accompanying rise in cardiovascular risk was noted (RR: 1.26; 95% CI: 0.49–3.23). Upadacitinib treatment, specifically, caused the strongest increment in LDL-C levels (RR: 17.22; 95% CI: 8.02–26.42), while it also increased HDL-C levels (RR: 6.78; 95% CI: 5.43–8.12) [42].

Previous meta-analyses have shown that JAKinibs, in general, are not associated with an increased cardiovascular risk (RR: 0.73; 95% CI: 0.22–2.43 and RR: 0.80; 95% CI: 0.36–1.75) [43,44]. It has to be noted that these meta-analyses included a smaller number of upadacitinib-related studies compared to the present one. What is more, in both of these studies, despite the lack of statistical significance, there was a slight tendency towards increased MACE in the upadacitinib groups, contrary to the other JAK inhibitors. Our study, however, shows that upadacitinib only carries a neutral cardiovascular risk, similar to that of other JAK inhibitors. In this meta-analysis, the follow-up of the included studies (12 to 52 weeks) was inadequate for a decisive characterization of cardiovascular risk. Notably, however, a recent post-authorization, noninferiority, open-label RCT (*n* = 4362, follow-up for 4 years) that compared tofacitinib (5 mg or 10 mg twice daily) with a tumor necrosis factor inhibitor (adalimumab or etanercept) demonstrated a higher risk of MACE in the case of tofacitinib (hazard ratio, 1.33; 95% CI, 0.91 to 1.94). Importantly, that study used a cardiovascular-risk-enriched sample and raised suspicions regarding the potential cardiovascular hazards of other JAKinibs as well [45].

To understand the effects of upadacitinib and other JAKinhibs on lipoproteins, one must first attempt to grasp the pathophysiological mechanisms linking inflammation to hyperlipidemia. Hyperlipidemia is encountered in multiple autoimmune and rheumatic diseases, including RA, spondyloarthritides, vasculitides, and systemic lupus erythematous. Its proposed mechanism involves changes in lipoproteins as part of the innate immune response [46,47]. This phenomenon is beneficial during acute inflammation, but in the case of chronic inflammation, it becomes detrimental and can lead to atherogenesis [47]. Hypercholesterolemia, itself, further enhances inflammatory responses via the accumulation of lipids in macrophages and other immune cells [48]. In states of systemic inflammation, especially RA, an increased cardiovascular risk is often present despite low levels of total cholesterol and LDL-C [49,50]. Conversely, when these patients receive specific treatment, lipoprotein levels increase, but the cardiovascular risk decreases [49,50]. Therefore, the upadacitinib-related increase in LDL-C levels may simply reflect the decrease in inflammation. This effect may explain the potentially protective cardiovascular effects of anti-rheumatic drugs such as JAK inhibitors in cases of RA, despite the elevation in LDL-C levels [49]. The increase in HDL-C may also reflect the anti-inflammatory effect of upadacitinib [49]. On the other hand, a study has shown that upadacitinib therapy increased LDL-C in patients with atopic dermatitis, a typical inflammatory skin disease [21,51]. The corresponding LDL-C increase was significant only in the 30 mg group [21]. This finding points to an alternative mechanism of hypercholesterolemia which does not rely solely on the resolution of inflammation.

When interpreting the results of the present study, one should exert caution, considering certain limitations. Some of these limitations derive from the short follow-up periods, the young age of the study participants (mean age of 51 years), the heterogeneity of the included disease populations, the small number of MACE, the paucity of reported cardiovascular disease risk factors, and the lack of adjustment for disease-related risk factors, such as the disease activity. Moreover, there was a lack of reporting on sequential lipoprotein measures, as well as the use of lipid-lowering agents. Additionally, a high heterogeneity was noticed in the meta-analysis regarding the effect of upadacitinib on LDL-C (I^2^ = 91%, *p* < 0.0001), but this can be attributed to differences in population characteristics between the three included studies. Specifically, two of the studies (McInnes et al. and Mease et al.) were conducted on patients with PsA [17,20], while Fleischmann et al. enrolled RA patients [22]. Moreover, this meta-analysis did not take into consideration the effects of concurrent treatments, such as disease-modifying anti-rheumatic drugs (DMARDs). Interestingly, according to a sub-analysis by Navarro-Millan et al., DMARDs were associated with an average increase of 30 mg/dL in the LDL-C after 6 months of therapy across all arms, i.e., methotrexate monotherapy, triple therapy (methotrexate, sulfasalazine, hydroxychloroquine), or methotrexate plus etanercept [52]. The incomplete reporting of data in a small number of studies might have also increased the bias risk. Lastly, the performance of multiple comparisons may introduce type I error. In the present review, this could have potentially occurred due to the existence of multiple outcomes or the subgroup analysis. However, this risk was minimized, as the outcomes were divided into primary (mean HDL-C and LDL-C changes) and secondary (MACE) groups, while the subgroup analysis was restricted to the clinically important dose of 15 mg alone.

The present review included the largest sample of upadacitinib-treated patients to date. Other strengths of this study include its generalizability, owing to the enrollment of patients with different diseases, as well as the concordance of the results, as evidenced by the low heterogeneity between the studies.

## 5. Conclusions

Treatment with upadacitinib increases both LDL-C and HDL-C levels, whereas the LDL-C:HDL-C ratio remains unaltered. Available evidence derived from short-term placebo-controlled RCTs shows that upadacitinib has no effect on the risk of MACE. In this context, further data derived from large prospective cohorts or specifically designed RCTs are required to fully address the cardiovascular safety of upadacitinib therapy.

## Figures and Tables

**Table 1:** Characteristics of the eligible studies.

First Author	Year	Country	RCT Phase	Population	Sample	Duration (Weeks)	Intervention			Comparator		
							Upadacitinib	Sample	Age	Arm	Sample	Age
Burmester [16]	2018	Multicentre	III	RA	661	12	15, 30 mg QD	440	55.5	Placebo	221	56.0
Deodhar [24]	2022	Multicentre	III	nr-axSpA	313	14	15 mg QD	156	41.6	Placebo	157	42.5
Fleischmann [14]	2019	Multicentre	III	RA	1629	48	15 mg QD	651	54.0	Placebo or ADA	978	54.0
Fleischmann [22]	2019	Multicentre	III	RA	1629	26	15 mg QD	651	54.0	Placebo or ADA	978	54.0
Genovese [9]	2016	Multicentre	IIb	RA	299	12	3–18 mg BID, 24 mg QD	249	55.0	Placebo	50	55.0
Genovese [15]	2018	Multicentre	III	RA	498	24	15, 30 mg QD	329	56.8	Placebo	169	57.6
Guttman-Yassky [21]	2020	Multicentre	IIb	AD	167	16	7.5–30 mg QD	126	40.0	Placebo	41	39.9
Heijde [12]	2019	Multicentre	II/III	AS	187	14	15 mg QD	93	47.0	Placebo	94	43.7
Heijde [25]	2022	Multicentre	III	AS	420	14	15 mg QD	211	42.6	Placebo	209	42.2
Kameda [11]	2020	Multicentre	IIb/III	RA	197	12	7.5–30 mg QD	148	55.5	Placebo	49	54.3
Kremer [8]	2016	Multicentre	IIb	RA	276	12	3–12 mg QD, 18 mg BID	220	57.2	Placebo	56	58.0
McInnes [17]	2021	Multicentre	III	PsA	1704	24	15, 30 mg QD	852	50.7	Placebo or ADA	852	50.9
Mease [20]	2021	Multicentre	III	PsA	641	24	15, 30 mg QD	429	53.0	Placebo	212	54.1
Reich [23]	2021	Multicentre	III	AD	901	16	15–30 mg QD	597	34	Placebo	304	34.3
Rubbert-Roth [18]	2020	Multicentre	III	RA	612	24	15 mg QD	303	55.3	Abatacept	309	55.8
Sandborn [10]	2020	Multicentre	II	CD	220	52	3–24 mg BID, 24 mg QD	183	42.2	Placebo	37	40.0
Smolen [19]	2019	Multicentre	III	RA	648	14	15, 30 mg QD	432	53.8	MTX	216	55.3
van Vollenhoven [13]	2020	Multicentre	III	RA	945	24	15,30 mg QD	631	53.4	MTX	314	53.3
Zeng [26]	2021	Multicentre	III	RA	338	12	15 mg QD	169	51.7	Placebo	169	51.7

AD: atopic dermatitis; ADA: adalimumab; AS: ankylosing spondylitis; CD: Crohn’s disease; MTX: methotrexate; nr-axSpA: non-radiographic axial spondyloarthritis; PsA: psoriatic arthritis; RA: rheumatoid arthritis BID: twice a day; QD: every day.

**Table 2:** Effects of upadacitinib on LDL-C and HDL-C in the eligible studies.

Study	Year	Disease	Duration (Weeks)	Intervention					Comparator				
				Upadacitinib	No	Outcomes (Mean Changes) *			Arm	No	Outcomes (Mean Changes) *		
						LDL-C	HDL-C	LDL-C: HDL-C			LDL-C	HDL-C	LDL-C: HDL-C
Burmester [16]	2018	RA	12	15 mg QD	221	12.3%	18.4%	0.116	Placebo	221	0.14%	1.04%	−0.04
				30 mg QD	219	15.3%	15.1%	0.006					
Deodhar [24]	2022	nr-axSpA	14	15 mg QD	156	N/A	N/A	N/A	Placebo	157	N/A	N/A	N/A
Fleischmann [14]	2019	RA	48	15 mg QD	651	19.61	6.3	N/A	Adalimumab	327	7.42	1.66	N/A
									Placebo	651	N/A	N/A	N/A
Fleischmann [22]	2019	RA	26	15 mg QD	651	19.84 ^a^	7.88	0.04	Adalimumab	327	2.44	0.54	0.016
									Placebo	651	−1.93	0.62	N/A
Genovese [9]	2016	RA	12	3 mg BID	50	10.828 ^a^	4.254	N/A	Placebo	50	−2.32	0.386	N/A
				6 mg BID	50	13.534 ^a^	6.574 ^a^						
				12 mg BID	50	18.95 ^b^	5.027						
				18 mg BID	50	10.44 ^a^	5.027						
				24 mg QD	49	11.214 ^b^	4.64						
Genovese [15]	2018	RA	24	15 mg QD	164	W12 14.617	W12 9.01	0.035	Placebo	169	−2.127	−0.734	N/A
						W24 13.34	W24 8.623		W12 Placebo to UPA 15 mg		0.206	9.667	0.027
				30 mg QD	165	W12 17.595	W12 13.264	0.002	W24 Placebo to UPA 30 mg		0.283	15.352	−0.04
						W24 19.335	W24 12.606						
Guttman-Yassky [21]	2020	AD	16	7.5 mg QD	42	0.154	4.099	−0.196	Placebo	41	−2.475	2.668	0.009
				15 mg QD	42	5.8	4.95	−0.008					
				30 mg QD	42	13.805 ^a^	12.065 ^b^	−0.11					
Heijde [12]	2019	AS	14	15 mg QD	93	12.297	10.17	−0.153	Placebo	94	−3.209	0.386	−0.066
Heijde [25]	2022	AS	14	15 mg QD	211	N/A	N/A	N/A	Placebo	209	N/A	N/A	N/A
Kameda [11]	2020	RA	12	7.5 mg QD	49	17.63	10.65	0.01	Placebo	49	3.68	−0.78	0.052
				15 mg QD	49	13.92	13.46	−0.068					
				30 mg QD	50	15.32	14.19	−0.03					
Kremer [8]	2016	RA	12	3 mg QD	55	−1.28	4.29	−0.15	Placebo	56	−1.04	−0.47	0.02
				6 mg QD	55	18.33	10.05	0.05					
				12 mg QD	55	21.12	8.7	0.14					
				18 mg BID	55	18.75	8.16	0.006					
McInnes [17]	2021	PsA	24	15 mg QD	429	15.777 ^a^	8.623	0.486	Placebo	423	0.966	0.116	0.131
				30 mg QD	423	17.285	9.629	0.587	Adalimumab	429	1.314	3.093	0.055
Mease [20]	2021	PsA	24	15 mg QD	211	8.469 ^a^	7.695	−0.14	Placebo	212	0.116	−0.309	0.01
				30 mg QD	218	17.517	9.397	0.049					
Reich [23]	2021	AD	16	15–30 mg QD	597	N/A	N/A	N/A	Placebo	304	N/A	N/A	N/A
Rubbert-Roth [18]	2020	RA	24	15 mg QD	303	14.308	7.734	0	Abatacept	309	1.933	1.933	−0.031
Sandborn [10]	2020	CD	52	3 mg BID	39	W16 1.93	W16 5.027	N/A	Placebo	37	W16 −0.386	W16 −0.773	N/A
						W52 5.8	W52 3.093						
				6 mg BID	37	W16 5.8	W16 1.93						
						W52 18.95	W52 8.12						
				12 mg BID	36	W16 ^b^ 16.628	W16 ^b^ 5.8 ^c^						
						W52 18.95	W52 8.12						
				24 mg BID	36	W16 ^b^ 16.24	W16 18.56						
						W52 N/A	W52 N/A						
				24 mg QD	35	W16 7.734	W16 0.0003						
						W52 11.214	W52 12.374						
Smolen [19]	2019	RA	14	15 mg QD	217	13.612	10.828	−0.055	MTX	216	0.038	0.116	−0.015
				30 mg QD	215	16.976	10.286	−0.042					
van Vollenhoven [13]	2020	RA	24	15 mg QD	317	18.95	8.894	N/A	MTX	314	2.32	1.16	N/A
				30 mg QD	314	27.84	12.76						
Zeng [26]	2021	RA	12	15 mg QD	169	N/A	N/A	N/A	Placebo	169	N/A	N/A	N/A

* Mean changes are expressed in mg/dL, unless percentages are shown. AD: atopic dermatitis; AS: ankylosing spondylitis; CD: Crohn’s disease; MTX: methotrexate; N/A: not applicable; nr-axSpA: non-radiographic axial spondyloarthritis; PsA: psoriatic arthritis; RA: rheumatoid arthritis; BID: twice a day; QD: every day; ^a^ *p* < 0.05 for the comparison with the placebo group; ^b^ *p* < 0.01 for the comparison with the placebo group; ^c^ *p* < 0.001 for the comparison with the placebo group.

## Data Availability

Not applicable.

## Supplementary Materials

The following supporting information can be downloaded at: https://www.mdpi.com/article/10.3390/jcm11236894/s1, Supplementary Table S1. Inclusion and exclusion criteria according to PICOS; Supplementary Table S2. Reasons for exclusion.
