# Supplementary material for: The Effect of Upadacitinib on Lipid Profile and Cardiovascular Events: A Meta-Analysis of Randomized Controlled Trials
Source: J Clin Med. 2022 Nov 22;11(23):6894. doi: 10.3390/jcm11236894 (PMC9740350; doi:10.3390/jcm11236894)
Supplement: Supplementary file 1 [file jcm-11-06894-s001.zip › jcm-2001700-supplementary.pdf]

The Effect of Upadacitinib on Lipid Profile and Cardiovascular Events:

A Meta-analysis of Randomized Controlled Trials

Supplementary Material

**Supplementary Table S1.** Inclusion and exclusion criteria according to PICOS		
	**Inclusion Criteria**	**Exclusion Criteria**
**Population**	All patients receiving upadacitinib	Patients not receiving upadacitinib
**Intervention**	Administration of upadacitinib, alone or in conjunction with background treatment	Other interventions, including other JAK inhibitors
**Comparator**	Placebo, or any other intervention	Does not apply
**Outcome**	Effect on lipid profile (LDL-C, HDL-C, LDL-C:HDL-C) and/or major adverse cardiovascular events (i.e., stoke, cardiovascular death, myocardial infarction).	Other outcomes, or lack of data regarding specific lipid variables of interest (i.e., LDL-C, HDL-C, LDL-C:HDL-C and major adverse cardiovascular events).
**Study Design**	Only randomized control trials	Other study designs

Supplementary Table S2. Reasons for exclusion	
**Study**	**Reasons for exclusion**
Silverberg et al., 2021	No data on lipoproteins, no data on MACE for control group
Deodhar et al., 2021	No data on lipoproteins, no data on MACE for control group
Fleischmann et al., 2022	No data on lipoproteins, no data on MACE for control group
Heijde et al., 2022	No data on lipoproteins, no data on MACE for control group
McInnes et al., 2021	No data on lipoproteins, no data on MACE for control group
Blauvelt et al., 2021	No data on lipoproteins, no data on MACE for control group
Mease et al., 2021	No data on lipoproteins, no data on MACE for control group
Kameda et al., 2021	No data on lipoproteins, no data on MACE for control group
Sandborn et al., 2020	No data on lipoproteins or MACE
Vermeire et al., 2021	No data on lipoproteins or MACE
Pavelka et al., 2020	Sub-analysis of identified trial
Yamaoka et al., 2021	Sub-analysis of identified trial
NCT02049138 (BALANCE-EXTEND)	Sub-analysis of identified trial
Danese et al., 2021	Identical to identified trial
